# Association between the hemodialysis adequacy and sexual dysfunction in chronic renal failure: a preliminary study

**DOI:** 10.1186/1471-2490-14-4

**Published:** 2014-01-08

**Authors:** Jae Heon Kim, Seung Whan Doo, Won Jae Yang, Soon Hyo Kwon, Eun Seop Song, Hong Jun Lee, Ik Sung Lim, Hyun Hwang, Yun Seob Song

**Affiliations:** 1 Department of Urology, Soonchunhyang University Hospital, Soonchunhyang University College of Medicine, Seoul, Korea; 2 Department of Nephrology, Soonchunhyang University Hospital, Soonchunhyang University College of Medicine, Seoul, Korea; 3 Department of Obstetrics and Gynecology, Inha University School of Medicine, Incheon, Korea; 4 Medical Research Institute, Chung-Ang University College of Medicine, Seoul, Korea; 5 Department of Industrial Management and Engineering, Namseoul University College of Engineering, Cheonan, Korea; 6 North London Collegiate School, Jeju, Korea

**Keywords:** Hemodialysis, Hemodialysis adequacy, Urea clearance, Female, Sexual dysfunction

## Abstract

**Background:**

The core question of the study was whether adequately achieved HD affected the sexual dysfunction in women on hemodialysis (HD) with chronic renal failure (CRF).

**Methods:**

Thirty-seven female patients on HD, including 18 women with adequate HD and 19 women with non-adequate HD, and 36 healthy controls were included in this study. Demographic and clinical variables, including the sexual hormones estradiol and testosterone, were recorded. Sexual function was assessed according to the Female Sexual Function Index (FSFI) and results were compared between groups. Adequate HD was defined as an average urea clearance of over 1.3 (Kt/V) over three consecutive months.

**Results:**

All domains of the FSFI questionnaire, with the exception of satisfaction, were higher in the control group than in the HD group. In comparing the adequate and non-adequate HD groups, there was no difference in any of the six domains of the FSDI questionnaire. Among the clinical variables, the number of menopausal women was higher in the HD group than in the control group (*P* = 0.023). Estradiol and testosterone levels were higher in the control group than in the HD group (*P* = 0.003, 0.027, respectively). The number of menopausal women and estradiol and testosterone levels showed no differences between the adequate and non-adequate HD groups. Correlation analysis between Kt/V and FSFI showed no significant relationship, but estrogen did show a significant relationship with FSFI (correlation coefficient = 0.399, *P* = 0.001)*.*

**Conclusions:**

HD adequacy alone does not have a significant impact on sexual dysfunction. Other treatments options should be considered to treat sexual dysfunction in women with CRF.

## Background

Female sexual dysfunction is common in women experiencing chronic renal failure (CRF) and is estimated to occur at a rate of 60-70% despite the use of dialysis [1,2].

Hemodialysis (HD) is the most commonly used option for dialysis, and often patients on HD have no choice but to continue HD until they undergo a kidney transplant (KT). Although there have been many reports regarding the improvement of sexual dysfunction after KT [3,4], those outcomes are not consistent [5,6], and moreover, from the patients’ perspective, the chance for a KT is not likely.

In HD patients, adequate HD is important because it enables the patient to live clinically asymptomatic and be reasonably active and to maintain correction of the altered metabolic and homeostatic components secondary to the loss of the kidney function [7]. Ultimately, adequacy of HD is related to reduced morbidity and mortality associated with CRF [7].

Urea clearance (Kt/V) is a fractional clearance and represents HD adequacy [8]. However, few studies have investigated whether HD adequacy can improve sexual function in women with CRF. The aim of this study was to assess the impact of HD adequacy on sexual dysfunction in women with CRF and on HD.

## Methods

### Study sample

From March 2008 to February 2011, a total of 37 consecutive married women with CRF who were on HD were eligible and willing to participate in the study. Healthy female volunteers (*n* = 36) from the health promotion center at Soonchunhyang University Hospital were included as controls.

Approval for this study was obtained from the Internal Review Board at Soonchunhyang University Hospital. The HD group was composed of patients receiving HD treatment three times a week for 4 hours at one dialysis procedure for at least 6 months at the HD center at Soonchunhyang University Hospital.

**Figure 1:**
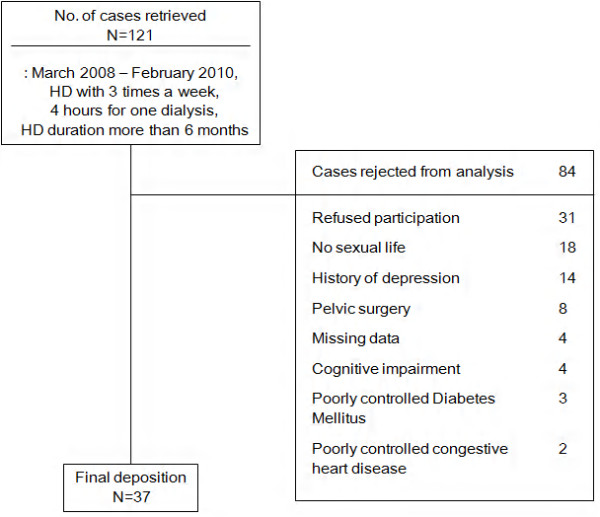
Patient deposition.

The eligibility criteria for inclusion were: between the ages of 18 and 60 years, female gender, married, sexually active, no psychiatric disease including depression in the previous 6 months, and the intellectual and mental capacity to understand and answer the questionnaire. Exclusion criteria were having undergone surgical menopause, having undergone pelvic surgery including hysterectomy, clinical depression or other major psychiatric disease and having used hormonal replacement therapy within the past 5 years. A total of 37 patients were included in this study (Figure 1).

### Clinical investigation

All patients and controls provided informed consent and underwent detailed clinical examination including testing hemoglobin, testosterone and estradiol levels, and recording of urea clearance (Kt/V).

To limit the influence of fluctuations in plasma hormone levels, blood samples were always drawn at the same time of day (08:00 to 10:00 hours).

### Methodology

This was a cross-sectional study. A single investigator conducted face-to-face interviews with all study participants using a structured questionnaire.

### FSFI questionnaire

To obtain sexual function assessments, the patients were asked to answer the Female Sexual Function Index (FSFI) questionnaire after undergoing HD treatment. The FSFI is an instrument used for the assessment of sexual function and consists of 19 questions [9,10].

The FSFI has been validated based on the DSM IV diagnoses of desire disorder, arousal disorder, and orgasmic dysfunction, and is intended for patients that have been sexually active in the previous four weeks [9,10]. The FSFI been previously validated in the Korean language and in the Korean population [11]. The questions are grouped and scored for domains of desire (two questions), arousal (four questions), lubrication (four questions), orgasm (three questions), satisfaction (three questions), and pain (three questions). Each domain is scored on a scale of 0 to 6, with higher scores indicating better function for each domain. A domain score of zero indicates that the women reported no sexual activity during the previous month and the full score ranges from 2 to 36. The individual domain scores are totaled and multiplied by a predetermined factor to weigh each domain equally.

### Assessment of urea clearance (Kt/V)

Urea clearance (Kt/V) is a fractional clearance and is indicative of HD adequacy. [8] Kt/V is calculated using the following natural logarithm formula: Kt/V = - Ln (R - 0.008 × t) + (4 – 3.5 × R) × UF/W, where Ln is the natural logarithm, R is the ratio between post-dialysis blood urea nitrogen (BUN) and pre-dialysis BUN, t is the dialysis session length in hours, UF is the ultrafiltration volume in liters, and W is the patient’s post-dialysis weight in kilograms.

### Definition of adequate HD

Adequate HD was defined as over 1.3 (Kt/V) average urea clearance over three consecutive months. The Kidney Disease Outcomes Quality Initiative (KDOQI) guidelines (2006) recommend that the Kt/V dose targets are 1.4 with a 1.2 minimum, and the Renal Association Clinical Practice guidelines recommend a single pool Kt/V of greater than 1.3.

### Statistical analyses

The Kolmogorov-Smirnov test was used to verify the normality of the distribution of continuous variables. Nonparametric comparison tests were used for variables evaluated as not normally distributed. Scores from the FSFI showed a nonparametric distribution; therefore, the mean value with standard deviation and the median value were used together as appropriate to describe statistics. Differences between groups were determined using the Kruskal-Wallis test and the Mann–Whitney test as appropriate. Spearmann’s correlation test was used to investigate the association between Kt/V and sexual functional scores. All statistics were two-tailed and a *P* value < 0.05 was considered significant. All calculations were performed using SPSS version 18.0 (SPSS, Chicargo, Ill., USA).

## Results

### Baseline analysis among the adequate HD, non-adequate HD, and normal control groups

There were no differences in age and height between the control and patient groups. Body weight and body mass index were significantly lower in the CRF patient group. The underlying etiologies of renal failure were chronic glomerulonephritis (*n* = 6), diabetic nephropathy (*n* = 12), hypertensive nephropathy (*n* = 6), adult polycystic kidney (*n* = 1) and other etiologies (*n* = 11). The mean duration of HD was 8.1 years.

Demographic variables did not differ, with the exception of body mass index, which was higher in the control groups. The rate of menopause was significantly higher in the HD groups, and testosterone and estradiol showed higher levels in the control groups. The average single–pool Kt/V in the adequate and non-adequate HD groups was 1.47 ± 0.11 and 1.14 ± 0.05, respectively (Table 1).

**Table 1:** Patient characteristics

	**Controls (*n*** **= 36)**	**Adequate HD (*n*** **= 18)**	**Non-adequate HD (*n*** **= 19)**	***P *** **value**
Age (years)	48.19 ± 6.94	47.89 ± 6.82	47.53 ± 6.39	0.671
Height (cm)	155.65 ± 5.46	155.61 ± 4.07	155.741 ± 4.50	0.978
Weight [24]	58.84 ± 7.27	56.11 ± 9.46	54.18 ± 5.76	0.052
Body mass index (kg/m^2^)	24.31 ± 3.02	23.08 ± 3.26^†^	22.37 ± 2.60^‡^	0.036^*^
Educational level				0.321
Middle school or less	8 (22.2%)	3 (16.6%)	3 (15.7%)	
High school	10 (27.7%)	10 (55.5%)	9 (47.3%)	
College or more	18 (50.0%)	5 (27.7%)	7 (36.8%)	
Monthly income (won)				0.072
<1 million	7 (19.4%)	6 (33.3%)	7 (36.8%)	
1-3 million	19 (52.7%)	7 (38.8%)	8 (42.1%)	
>3 million	10 (27.7%)	5 (27.7%)	4 (21.0%)	
Menopause (%)	10 (27.7%)	13 (66.5%)	12 (63.1%)	0.023^**^
Hematocrit (%)	29.8	28.1	28.3	0.057
Testosterone (ng/ml)	1.25 ± 2.25	0.45 ± 0.06^†^	0.26 ± 0.08^‡^	0.027^*^
Estradiol (pg/ml)	80.93 ± 77.24	27.55 ± 20.82^†^	34.68 ± 55.69^‡^	0.003^*^
Duration of HD (years)		7.4 ± 6.99	8.5 ± 3.99	0.781
Onset of CRF		10.5 ± 5.29	11.5 ± 6.72	0.574
Average single-pool Kt/V		1.47 ± 0.11	1.14 ± 0.05	0.021^***^

HD, hemodialysis, CRF, chronic renal failure. ^*^analyzed by Kruskal-Wallis test; ^**^analyzed by Fisher’s exact test; ^***^analyzed by Mann Whitney test. ^†^Control versus adequate HD, ^‡^Control versus non-adequate HD.

### Comparative analysis of FSFI scores among the adequate HD, non-adequate HD, and normal control groups

When comparing the FSFI scores in the control and study groups, all domain scores except ‘satisfaction’, namely ‘desire’, ‘arousal’, ‘lubrication’, ‘orgasm’, ‘pain’ and ‘total’, were significantly lower in the HD groups than the scores of the control groups. The adequate and non-adequate groups did not differ in any of the domains or total FSFI (Table 2).

**Table 2:** Female sexual function index in patients with chronic renal failure who are on hemodiaysis

**FSFI**	**Controls (*n*** **= 36)**	**Adequate HD (*n*** **= 18)**	**Non-adequate HD (*n*** **= 19)**	***P *** value^*^
Desire	2.4 ± 0.90, 2.4	1.43 ± 0.68^†^, 1.2	2.08 ± 2.47^‡^, 1.2	<0.001
Arousal	2.68 ± 1.90, 3.0	1.11 ± 1.71^†^, 0	0.93 ± 1.87^‡^, 0	0.001
Lubrication	3.30 ± 2.33, 3.9	1.66 ± 2.44^†^, 0	1.16 ± 2.33^‡^, 0	0.004
Orgasm	2.88 ± 2.11, 3.6	1.48 ± 2.23^†^, 0	0.96 ± 1.93^‡^, 0	0.004
Satisfaction	3.45 ± 1.66, 4.0	2.06 ± 1.70, 0.8	2.88 ± 1.79, 4.0	0.052
Pain	3.37 ± 2.27, 4.0	1.75 ± 2.59^†^, 0	1.20 ± 2.40^‡^, 0	0.011
Total	18.11 ± 10.24, 22.0	9.52 ± 10.64^†^, 2	8.76 ± 9.52^‡^, 5.2	0.003

Values are expressed as mean ± SD, median value.

HD, hemodialysis; FSFI, Female Sexual Function Index. ^*^analyzed by Kruskal-Wallis test.

^†^Control versus adequate HD, ^‡^Control versus non-adequate HD.

### Correlation analysis between Kt/V and FSFI scores

Spearman’s correlation test was performed to evaluate the associations among the FSFI questionnaire and Kt/V. The correlation analysis showed no significant relationship between FSFI and Kt/V (Table 3).

**Table 3:** Correlation coefficients between average single-pool Kt/V and female sexual function index score

**FSFI**	**Correlation coefficients urea clearance**	***P *** value^*^
Desire	-0.156	0.355
Arousal	0.194	0.250
Lubrication	0.194	0.251
Orgasm	0.243	0.147
Satisfaction	-0.113	0.504
Pain	0.227	0.177
Total	-0.052	0.761

FSFI, Female Sexual Function Index. ^*^analyzed by Spearman’s correlation test.

### Correlation analysis between other factors and FSFI scores

Age, duration of dialysis, body mass index and testosterone showed no significant correlation with FSFI scores. Estrogen showed a significant correlation with all FSFI domains: ‘desire’ (*P* < 0.001), ‘arousal’ (*P* < 0.001), ‘lubrication’ (*P* < 0.001), ‘orgasm’ (*P* < 0.001), ‘satisfaction’ (*P* = 0.047), ‘pain’ (*P* = 0.003), and ‘total FSFI’ (*P* = 0.001).

## Discussion

Female patients with CRF often suffer from sexual dysfunction [1,4]. Sexual dysfunction in women with CRF is largely due to loss of sexual interest, difficulties with arousal and reaching orgasm, reduced libido and lubrication, and pain during intercourse [1,12,13].

Considering that there are no other treatment options except KT, maintaining HD is an important treatment strategy for patients with CRF. HD adequacy is directly related to a patient’s well–being, including physical activity, and is directly related to mortality and morbidity [7]. Considering there is no standard protocol for treating sexual dysfunction in women with CRF on HD, one important motivation for this study arose from the need to investigate the impact of HD adequacy on female sexual dysfunction.

Kt/V, urea fractional clearance, is a standard method to assess HD adequacy. This kinetic model is a clear was of determining adequacy [8]. There have been few reports regarding the role of Kt/V in female sexual dysfunction in patients with CRF [14,15]. Previous studies reported that HD adequacy was not related to female sexual dysfunction. One of the limitations of those studies was the application of the Kt/V results. Recently, the recommended dose was increased to a target of 1.4. The KDOQI guidelines (2006) recommend a Kt/V dose target of 1.4 with a minimum of 1.2, and the Renal Association Clinical Practice guidelines recommend a single pool Kt/V of greater than 1.3. More importantly, HD adequacy cannot determined by a single Kt/V, but rather consecutive and consistent target doses of Kt/V are more important. In our study, adequate HD was defined as an average Kt/V of 1.3 over three consecutive months.

Although our study did not find an association between HD adequacy and female sexual dysfunction, this is the first study to assess the relation between sexual dysfunction and HD adequacy as measured using the consecutive method in women with CRF.

We reported in a previous study that the score of all domains of the FSFI questionnaire, ‘desire’, ‘arousal’, ‘lubrication’, ‘orgasm’, ‘satisfaction’ and ‘pain’, were significantly lower in the patient group than in the control group [16]. However, it is not clear that HD adequacy is related to sexual dysfunction. Our results show that HD adequacy alone does not restore sexual dysfunction in women with CRF. This is in agreement with a report that sexual dysfunctions does not improve with dialysis treatment [17].

As the genesis of sexual dysfunction is multifactorial in CRF patients, one aspect of HD adequacy alone was insufficient for explaining sexual dysfunction. It is believed that the lack of estradiol-stimulated cyclic LH secretion in women on dialysis leads to ovarian failure, which is presumed to be the primary cause of infertility [13,15,18,19]. Our results showed that levels of testosterone and estradiol were significantly decreased in the patient group. Moreover, estradiol showed a significant relationship with the scores in all domains of the FSFI. A previous study demonstrated that hormone replacement therapy allows sustained physiological serum estradiol concentrations in women with estrogen deficiency undergoing HD, with an associated improvement in sexual function [20]. It is suggested that adequate treatment of multiple factors, including emotional derangement and sex hormone change, is also necessary for improvement in sexual function in female CRF patients. Among them, hormonal replacement therapy (HRT) could be a promising treatment option. To date, only 17% of dialysis women had ever been treated with HRT and even less (6%) were currently on such therapy [21]. The benefits of HRT in premenopausal women on dialysis with estrogen deficiency treated with transdermal HRT have been reported to show a sustained increase in estrogen and recovery of menstruation [20].

This study had some limitations. First, our patient population was quite small. Generally, the participation rate in sex research is very low and the degree of conservatism in sexual attitudes was very high in those who refused participation. Second, the patients represented only a small geographic area, which limits the generalization of our findings. Third, we did not investigate the depression quantification by questionnaire. Although we excluded those patients with depression, concurrent and subclinical depression could be determined through a questionnaire. However, the diagnostic cut-off values of the Beck depression inventory, which is one of most commonly used questionnaires to assess depression, is not consistent between the DSM-IV criteria and Korean validation form [22]. Therefore, the Beck depression inventory was not applied in our study. Fourth, we have not investigated other factors such as vascular abnormalities, medications, family interactions, and personal and social characteristics. Lastly, this study was not a prospective study. A prospective study of CRF patients is difficult as symptom onset is diverse, and indication and the method of dialysis differ according to each patient’s medical condition. Further research, including a multi-center prospective study, is warranted for investigating sexual dysfunction in females with CRF who are undergoing HD.

## Conclusion

In summary, sexual dysfunction was found in women with CRF who were on HD. HD adequacy alone does not have an impact on sexual dysfunction. Our results indicate that new strategies for the treatment of sexual dysfunction in women with CRF who are on HD are needed.

## Abbreviations

CRF: Chronic renal failure; HD: Hemodialysis; FSFI: Female sexual function index; HRT: Hormonal replacement therapy.

## Competing interest

The authors have no competing interest to disclose.

## Authors’ contributions

JHK and YSS contributed with the conception and design of the study and drafted the manuscript, SWD, SHK, ISL, and HH collected data and performed the analyses, WJY, ESS, and HJL assisted with conception and design of the study, conceived of the study and supervised the study and helped draft the manuscript. All authors read and approved the final manuscript.

## Pre-publication history

The pre-publication history for this paper can be accessed here:

http://www.biomedcentral.com/1471-2490/14/4/prepub
